# An outbreak investigation of *Legionella* non-*pneumophila* Legionnaires’ disease in Sweden, April to August 2018: Gardening and use of commercial bagged soil associated with infections

**DOI:** 10.2807/1560-7917.ES.2021.26.7.1900702

**Published:** 2021-02-18

**Authors:** Emma Löf, Fanny Chereau, Pontus Jureen, Sabina Andersson, Kristina Rizzardi, Petra Edquist, Sharon Kühlmann-Berenzon, Ilias Galanis, Caroline Schönning, Madeleine Kais, Anne Tideholm Nylén, Anders Wallensten, Adam Roth, on behalf of the National Outbreak Investigation Team

**Affiliations:** 1 Public Health Agency of Sweden (PHAS), Solna, Sweden; 2 European Programme for Intervention Epidemiology Training (EPIET), European Centre for Disease Prevention and Control, (ECDC), Stockholm, Sweden; 3 Stockholm County Council Department of Communicable Disease Prevention and Control, Stockholm, Sweden; 4 Members (other than the authors) of the National Outbreak Investigation Team are listed in the Acknowledgements.

**Keywords:** Legionnaires’ disease, Legionella non-pneumophila, Legionella longbeachae, outbreak, soil, case control study

## Abstract

In early June 2018, an increase in non-travel-related cases of *Legionella* non-*pneumophila* Legionnaires’ disease (LD) was observed in Sweden and a national outbreak investigation was started. Outbreak cases were defined as notified confirmed or probable cases of *L.* non-*pneumophila* LD, with symptom onset after 1 April 2018. From April to August 2018, 41 cases were reported, 30 of whom were identified as *L. longbeachae.* We conducted a case–control study with 27 cases and 182 matched controls. Results from the case–control study indicated that gardening and handling commercial bagged soil, especially dusty dry soil, were associated with disease. *L. longbeachae* was isolated in soils from cases’ homes or gardens, but joint analysis of soil and human specimens did not identify any genetic clonality. Substantial polyclonality was noted between and within soil samples, which made finding a genetic match between soil and human specimens unlikely. Therefore, whole genome sequencing may be of limited use to confirm a specific soil as a vehicle of transmission for *L. longbeachae.* Handling soil for residential gardening was associated with disease and the isolation of *L. longbeachae* in different soils provided further evidence for *Legionella* non*-pneumophila* infection from soil.

## Background

Legionellosis is caused by the Gram-negative bacterium *Legionella*. The severity of legionellosis varies from mild febrile illness (Pontiac fever) to a potentially fatal form of pneumonia (Legionnaires’ disease (LD)) [1]. Large outbreaks have been reported from many parts of the world [2]. Known risk factors for disease include increasing age, being male, smoking, diabetes mellitus, chronic lung disease, renal disease and compromised immunity [1,3].

The majority of reported cases of LD worldwide are caused by *Legionella pneumophila* [2] which is also the case in Sweden, where *L. pneumophila* accounted for 65–78% of reported domestic cases from 2015 to 2017 (data not shown). The *L.* non-*pneumophila* group contains many species, including *L. longbeachae* and *L. bozemanii,* which are infrequently reported causes of LD in Sweden. In Europe, only 1% of reported LD can be attributed to *L. longbeachae* [4]. In Australia and New Zealand, *L. longbeachae* is as common or more prevalent than *L. pneumophila* as a cause of LD [5-8].

In Sweden, as well as in the rest of Europe, the most common diagnostic test to detect *Legionella* in clinical specimens is the *L. pneumophila* serogroup 1 specific urinary antigen test (UAT). As additional diagnostic methods are needed to confirm other species of *Legionella* [9,10], these species are likely to be under-reported when only the UAT is used. When routine testing for *L. pneumophila* and *L. longbeachae* with PCR of lower respiratory specimens was introduced in New Zealand, a fourfold increase in annual LD cases was observed, with the majority caused by *L. longbeachae* [11].

Cases of *L. longbeachae* and *L. bozemanii* have been connected to use of potting soils, commercial bagged soils and compost materials, predominatly in Australia and New Zealand [12,13] but also in other low-incidence countries [14-17]. In Australia, health warning labels are present on bagged soil packages, informing users that the soil is a potential vehicle of microorganisms such as *L. longbeachae* and providing advice on how consumers can protect themselves to reduce the risk of contracting LD from soil products [12].

In Sweden, the first case of *L. longbeachae* was reported in 1987 [18] and only three to 13 cases have been reported annually since 2006. A small number of cases with *L. longbeachae* and *L. bozemanii* were previously suspected to be linked to soils and bagged soil; in 2011, a case contracted their *L. longbeachae* infection from occupational contact with soil containing *L longbeachae* and *L. bozemanii* [19].

### Outbreak detection

From April to June 2018, a threefold increase in *L.* non-*pneumophila* LD was observed at the national level in Sweden. In early June 2018, the Stockholm County Council Department of Communicable Disease Control and Prevention (Stockholm CDC department) undertook routine source tracing among an increasing number of domestic cases of *L.* non-*pneumophila* LD, many of whom reported being active gardeners. In June, *L. longbeachae* was identified by serology in four cases and by matrix-assisted laser desorption ionization time-of-flight mass spectrometry (MALDI-TOF MS) in one case, of 22 reported *L.* non-*pneumophila* cases. Therefore, an outbreak of *L.* non-*pneumophila*, including *L. longbeachae,* was suspected and investigated by a National Outbreak Investigation Team with the following objectives: (i) describe the magnitude of the outbreak, (ii) identify risk factors associated with infection with *L.* non-*pneumophila*, and (iii) ascertain, via environmental and laboratory investigation, any link between cases and a common species or strain of *L.* non-*pneumophila* causing the outbreak. Based on the results from these activities, the aim was to implement control measures in the case of a common source and/or develop national recommendations to address specific risk factors.

## Methods

### Epidemiological investigation

#### Outbreak case definition

For this study, outbreak cases were defined as *L.* non-*pneumophila* cases with symptom onset after 1 April 2018, reported to SmiNet (the Swedish electronic disease notification system) as probable or confirmed LD cases according to the Swedish case definition [20]. Cases were excluded if they reported a history of travel outside of Sweden within 14 days before onset of symptoms.

#### Description of cases

Demographic, laboratory, clinical and residential information on cases was retrieved from SmiNet. Cases were described by age (median and range), sex (proportion), county of residence and onset date. Incidence of cases by county was calculated using the 2017 population census by county [21] as denominator.

#### Case–control study

We conducted an individually matched case–control study, including cases with onset from 14 May 2018 (week 20).

Assuming response from 25 cases, probability of exposure of 0.5 among controls and correlation between cases and controls of 0.2, we required at least six controls per case in order to detect an odds ratio (OR) of 4 with 80% statistical power.

Controls were selected from Hälsorapport, a national panel at the Public Health Agency of Sweden (PHAS) consisting of 6,000 randomly selected individuals from the Swedish population. Controls were individually matched to each case based on county of residence, sex and age (± 5 years). Two online questionnaires, one for cases and one for controls, were developed by the PHAS and the Stockholm CDC department. Cases were asked about possible exposures within the 14-day period before onset of symptoms and controls were asked about exposures for the period of 14 days before the onset date of their corresponding matched case.

The case questionnaire collected information on symptoms (respiratory problems, cough, myalgia, fever, headache and diarrhoea) and hospitalisation for LD. Controls were asked about any episode of fever, cough or respiratory illness during the 14-day period. Cases and controls were queried about any underlying medical condition (heart problem, chronic respiratory disease, diabetes, chronic immune disease or other chronic conditions), smoking habits (current, past, never), hospitalisation and exposures related to specific gardening activities and use of gardening products. Controls were excluded if they had travelled abroad for the total 14-day period, been diagnosed with pneumonia or LD or if they had experienced cough and fever, or respiratory illness and fever, during the 14-day period before the onset date of their matched case.

Interviews were conducted by staff of the regional CDC departments. Only cases and controls who consented to interviews were included in the analysis.

#### Statistical analysis

As all included cases reported gardening during the exposure period of interest, we focused on identifying particular gardening activities and use of products that could be associated with illness. We tested the association between exposures and the risk of infection with *L*. non-*pneumophila* with conditional logistic regression, computed matched odds ratios (mOR) for each single exposure in univariable analysis and further assessed associations with adjusted mOR in multivariable analysis.

#### Products used for gardening

To investigate which products (bagged soil, bagged manure fertiliser, compost from own garden, bagged ground bark) were associated with *L.* non-*pneumophila* infection, we fitted a multivariable regression model applying backwards stepwise elimination at a p value of p > 0.05. Ground bark in bag was excluded from the model since only three cases reported using it. We tested whether being a current/former smoker or having any underlying conditions were effect modifiers in this model by including interaction.

#### Reported use of commercial bagged soil

As the previous model only included bagged soil, we further investigated specific use and characteristics of bagged soil (using dusty soil, spraying water on soil from bag, using soil in greenhouse, mixing soil with other products) in a multivariable model without elimination becasue the number of cases was small.

### Laboratory investigation

#### Laboratory confirmation of *Legionella* from human specimens in Sweden

Clinical hospital laboratories and regional laboratories used different diagnostic tests, such as UAT, PCR, culture and/or serology to identify and characterise *Legionella* spp. During the outbreak investigation, clinical laboratories were encouraged to send samples with a positive *L.* non-*pneumophila* PCR result to the regional laboratory in Jönköping for PCR identification of *L. longbeachae* using the Fast Track Diagnostics (FTD) respiratory pathogens 33 kit (Fast Track Diagnostics, Esch-sur-Alzette, Luxembourg) ) (FTD-PCR).

The laboratory at the PHAS received isolates for typing. Species characterisation was performed by MALDI-TOF MS and 16S-sequencing, and molecular epidemiology was analysed using whole genome sequencing (WGS) (described below).

Cases were classified as *L. longbeachae* or *L. bozemanii* infections if the results from the diagnostic tests (culture, 16S-sequencing, paired serology, FTD-PCR or serology with a single high titre) were positive for the respective species. This definition differs from the definition of a confirmed case used in Sweden for surveillance purposes, which defines *L.* non-*pneumophila* cases only as ‘probable’ cases if they are diagnosed with methods other than culture [20].

#### Laboratory confirmation of *Legionella* from environmental samples in Sweden

Soil samples from bagged soil and from pots were collected from cases’ homes and gardens by the municipal environmental health departments. Commercial laboratories cultured soil and water samples, according to the ISO standard 11731:1998 and ISO 11731:2017. As commercial laboratories generally can differentiate only between *L. pneumophila* and *L.* non-*pneumophila*, environmental isolates were sent to PHAS for species characterisation by MALDI-TOF MS and for molecular epidemiology by WGS, to enable comparison to the human isolates.

#### Whole genome sequencing and phylogenetic analysis

WGS was performed at PHAS using the Ion Torrent platform, aiming to read lengths of 200 bp and coverage of 20×. The sequence data were analysed by CLC Assembly Cell 5.1.1 software (Qiagen, Hilden, Germany). After mapping, variants were called, positions with low coverage (< 10) or ambiguous calls (< 90%) in one or more samples were filtered out. Single nucleotide polymorphisms (SNP) within 500 bp of each other were filtered out as single events from the SNPs in the phylogenetic analysis to remove nonrandomly distributed mutations; these were collectively termed recombination events. These mutations are considered a single event for the purpose of genetic distance. A minimum spanning tree was constructed on the basis of all the SNP differences.

### Ethical statement

Ethical approval was not necessary because the data used for the study were collected as part of the infectious disease surveillance defined by national legislation.

## Results

### Epidemiological investigation

#### Description of cases

**Figure 1:**
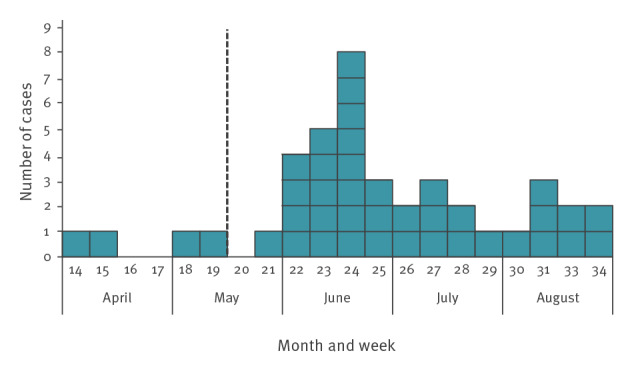
Distribution of cases diagnosed with *Legionella* non*-pneumophila* infection, by month and week of symptom onset^a^, Sweden, April–August 2018 (n = 41)

**Figure 2:**
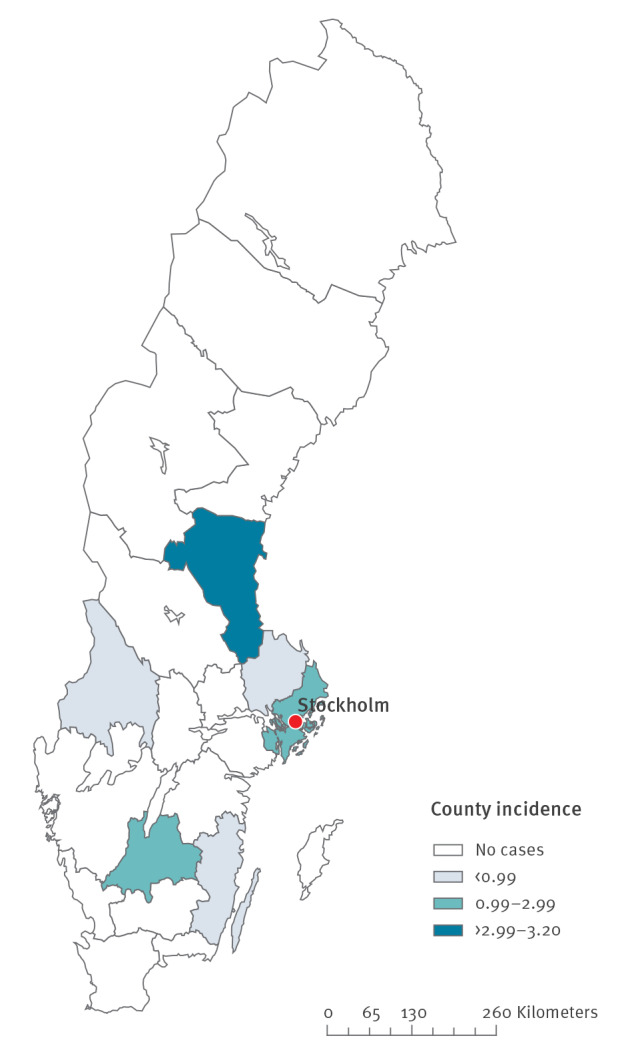
Incidence by county (cases/100,000 inhibitants) of cases diagnosed with *Legionella* non*-pneumophila* infection, Sweden, April–August 2018 (n = 41)

The outbreak investigation identified a total of 41 cases reported from April to August 2018. Two of the cases died within 30 days of symptom onset. The median age was 70 years (range: 46–87 years) and 20 of the cases were women. Symptom onset ranged from 3 April to 24 August 2018 (Figure 1) and cases were reported from six different counties (Figure 2), with the majority (n = 23) reported from Stockholm County.

The dashed verticle line indicates 14 May 2018, the start of eligibility for inclusion in the case–control study.

^a^Sampling date was used as date of onset for four cases for whom symptom onset was not available.

#### Case–control study

**Figure 3:**
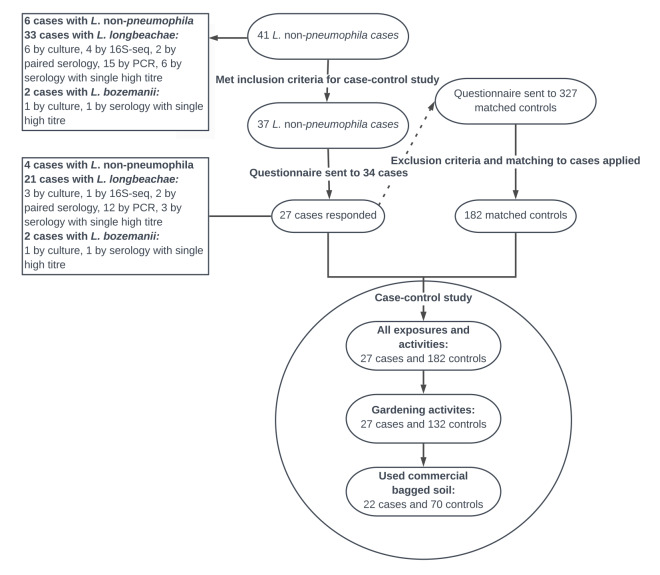
Selection of cases and controls and stepwise selection of variables for analysis in the *Legionella* non*-pneumophila* case–control study, Sweden, April–August 2018 (n = 41 cases)

Of the 37 *L.* non*-pneumophila* cases who met the inclusion criteria of symptom onset after 14 May 2018, 27 responded and could be included in the case–control study (three cases were not sent the questionnaire because of ongoing hospitalisation or because they were deceased). The median age was 69 years (range: 48–87 years) and 15 were women. After applying exclusion criteria on the controls, 182 controls were individually matched to the 27 cases. The inclusion and exclusion of cases and controls and the stepwise selection of the variables included in the regression models in the case–control study are shown in Figure 3.

##### Symptoms

Fever was reported by the majority (n = 26) of the cases, while the second most frequent symptom was headache (n = 14), followed by cough (n = 12), respiratory problems (n = 11), myalgia (n = 8) and diarrhoea (n = 7). All 27 cases were admitted to hospital.

##### Underlying medical conditions

The most frequent underlying condition among cases were immunosuppression (n = 9) and heart conditions (n = 9), followed by other chronic conditions (n = 8), respiratory conditions (n = 6) and diabetes (n = 3). The majority of cases (n = 23) suffered from at least one of these conditions. When compared with controls, cases were more likely to suffer from underlying immunosuppression (mOR = 10.6; 95% confidence interval (CI): 3.5–31.7), respiratory disease (mOR = 4.4; 95% CI: 1.4–13.4), heart conditions (mOR = 5.0 95% CI: 1.8–14.1), diabetes (mOR = 2.5; 95% CI: 0.6–9.7) and other underlying disease (mOR = 2.5; 95% CI: 1.0–6.4). Moreover, cases were more likely to have at least one of the conditions above (mOR = 13.5; 95% CI: 3.9–46.7)

##### Smoking

One case reported being an active smoker, 13 cases reported that they were former smokers, and the remaining 13 cases were non-smokers. Among the controls, 17 reported being a smoker, 32 controls were former smokers and the remaining controls were non-smokers. Cases were more likely than controls to be current or former smokers (mOR = 2.7; 95% CI: 1.2–6.0).

##### Common locations and activities among cases

The questionnaires did not identify a common location visited by the cases during the exposure period. All cases reported that they had been involved in gardening activities during the 14 days before symptom onset.

##### Reported gardening activities

All cases and 132 of the 182 controls reported gardening activities during the period of interest. In univariate analysis, four gardening products and 15 specific gardening activities were associated with illness with p values of p < 0.2 (Table 1).

**Table 1:** Gardening products used, product characteristics and gardening activities performed: Univariate matched odds ratio of exposures among cases diagnosed with *Legionella* non*-pneumophila* infection, Sweden, May–August 2018 (n = 27 cases, n = 132 controls)

Exposure variable (yes/no)	Number of cases		mOR	95% CI interval	p value
	Answered question	Exposed			
Products used					
Bagged ground bark	27	3	3.4	0.8–15.2	0.11
Bagged soil	27	22	2.6	0.9–7.5	0.09
Compost from own garden	27	7	2.6	0.9–7.4	0.08
Bagged manure fertiliser	27	12	2.3	0.9–5.8	0.08
Activities with products or characteristics of products					
Using dusty/dry bagged soil	27	5	23.1	2.7–199.5	0.001
Mixing bagged manure fertiliser with soil	12	10	6.2	0.7–56.6	0.11
Spraying water on soil from bag	22	16	5.7	1.1–29.0	0.04
Using bagged soil in greenhouse	22	4	5.4	0.9–31.1	0.06
Using bagged manure fertiliser in greenhouse	12	3	5.0	0.5–50.0	0.18
Gardening outdoors using garden soil	25	24	4.5	0.5–36.4	0.16
Using moist bagged mulch	27	2	4.2	0.6–29.9	0.16
Planting outdoors	25	22	2.8	0.8–10.7	0.12
Mixing bagged soil with other products	22	12	2.6	0.9–7.5	0.08
Mowing lawn	25	9	0.4	0.2–1.0	0.05
Watering hanging pots	25	5	0.3	0.1–1.0	0.05
Using bagged soil indoors	22	2	0.2	0.0–1.0	0.05
Spraying water on fertiliser	6	2	0.2	0.0–2.0	0.18
Using moist bagged soil	27	1	0.2	0.0–1.8	0.15
Tending indoor plants^a^	27	11	0.1	0.0–0.4	< 0.0001

CI: confidence interval; mOR: matched odds ratio.

^a^ Replanting, nurturing, watering etc.

##### Products used during gardening activities

In the multivariable model, analysing a combination of products used and the factors smoking and underlying disease, only bagged soil among the products was associated with LD (mOR = 2.6, 95% CI:0.9–7.5). Neither smoking nor any underlying conditions proved to be effect modifiers.

##### Reported use of commercial bagged soil

We further assessed all activities involving bagged soil among cases and controls who used bagged soil (22 cases, 70 controls). Cases had 15 times higher odds to have handled dusty/dry bagged soil compared with controls (Table 2).

**Table 2:** Adjusted matched odds ratios among cases diagnosed with *Legionella* non*-pneumophila* infection and controls, who reported use of commercial bagged soil, Sweden, April–August 2018 (n = 22 cases, n = 70 controls)

Exposure	Adjusted mOR	95%CI	p value
Using dusty bagged soil	15.0	1.2–185.9	0.04
Using bagged soil in greenhouse	3.5	0.5–25.1	0.21
Spraying water on soil from bag	4.0	0.6–25.6	0.14
Mixing bagged soil with other products	2.5	0.7–8.8	0.16

CI: confidence interval; mOR: matched odds ratio.

##### Brand of bagged soil, manure fertiliser or fertiliser

No single brand of bagged soil, compost, manure or other fertiliser could be confirmed to have been used by a majority of cases. The brands that cases reported to have used were provided by different distributors and producers on the Swedish market.

### Laboratory investigation

#### Case specimens

Forty-one domestic cases were laboratory-confirmed with *L.* non*-pneumophila* infections with onset between April and August 2018. For 35 of those, samples were available for further analysis by additional diagnostic methods such as culture, sequencing, PCR, FTD-PCR or serology; some cases therefore had results from multiple diagnostic tests. A total of 30 cases were classified as *L. longbeachae*; six by culture, four by 16S-sequencing, two by paired serology, 12 by FTD-PCR and six by serology with a single high titre. In total, two cases were classified as *L. bozemanii*; one by culture and one by serology with a single high titre.

#### Environmental specimens

The laboratory at PHAS received isolates from 21 different soil or fertiliser/manure samples from 16 case homes or gardens. From these, 11 samples were culture-positive for *L. longbeachae* and eight samples for *L. bozemanii*; in most samples, other *Legionella* species were also present.

#### Genetic diversity between human and environmental specimens

**Figure 4:**
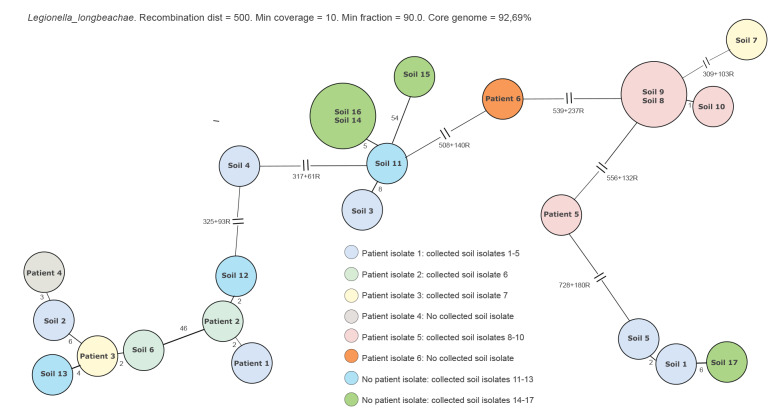
Minimum spanning tree of confirmed *Legionella longbeachae* from six cases and seven soil samples, Sweden, April–August 2018 (n = 6 case isolates, n = 17 soil isolates)

Human case isolates were available from six cases, four of these cases and two cases with no human case isolate aslo had soil samples available where isolates could be used for analysis by WGS. In total, six human case isolates and 17 environmental isolates (from seven different soil samples from homes or gardens of six cases) were analysed by WGS after being confirmed as *L. longbeachae* by MALDI-TOF MS. Isolates of *L. longbeachae* within a soil sample were diverse and mostly polyclonal. Furthermore, no isolate from a case and its corresponding soil sample isolates were identical. However, some genetic similarity could be seen between a few cases and also between cases and soil isolates from other cases’ homes or gardens (Figure 4).

Number of single nucleotide polymorphism differences are shown next to branches, recombination events are marked R. Length of branch is not indicative of relationship distance.

### Outbreak control measures

The regional CDC departments advised primary healthcare centres and hospitals that patients with pneumonia who are suspected to have LD could be infected with *L*. non-*pneumophila* which is not detectable by UAT, stressing the need for culture or PCR for detection and diagnosis.

Information about the outbreak was shared via the official website of PHAS, which emphasised specific measures to avoid inhalation of aerosols or dust from soil or compost materials. Press releases to national media were issued by the PHAS, the State Epidemiologist and Deputy State Epidemiologist as well as County Medical Officers (from regions with notified cases) participated in interviews for national television, radio and newspapers. Several national newspapers reported about the outbreak. While the outbreak investigation was closed on 31 August 2018, four additional suspected cases were reported from Stockholm County during the autumn.

## Discussion

During the spring and summer of 2018, Sweden experienced an increase in LD cases reported with *L.* non-*pneumophila* infection. The number of *L.* non-*pneumophila* cases was unusual compared with previous years and was investigated as an outbreak with the aim to implement control measures in the case of a common source and/or develop national recommendations to address specific risk factors. In the course of the investigation, *L. longbeachae*, a species that is rarely reported in Europe, was identified in 30 cases. This is one of the largest clusters of reported *L. longbeachae* LD cases in Sweden and, to our knowledge, in Europe. However, only six of these cases could be classified as confirmed *L. longbeachae* cases according to the European case definition used for surveillance purposes [22] and also according to the Swedish case definition [20], while the other 24 were classified as probable cases. The case–control study indicated that gardening and activities related to handling compost, bagged soil or soil products, particularly dusty dry soil, were associated with disease. *L. longbeachae* was isolated from bagged and garden soils from cases’ homes or gardens, supporting the hypothesis of infection by inhalation of contaminated dust from soils during gardening activities.

The WGS analysis of *L. longbeachae* isolates from six cases and of 17 isolates from seven soil samples revealed that cases’ isolates had different genetic profiles. Moreover, no soil isolate from cases’ homes or gardens was genetically identical to the corresponding case isolate. Interestingly, within the same soil sample, several isolates were polyclonal. Similar findings have also been noted by Bacigalupe et al. [23]. Our case–control study could not confirm that cases had used the same brand of bagged soil, compost or fertiliser. Given these combined results, we were unable to identify a single source of infection from soil or soil products. However, soil cannot be excluded as the source of infection considering the substantial polyclonality; the investigation did not allow analysis of all possible genetic variants and a link between soil and cases may have been missed even though multiple isolates from soil samples were analysed.

We have hypothesised that there might have been an increasing growth of *L.* non-*pneumophila* in bagged soil and further studies are needed to elucidate the baseline load of *L.* non-*pneumophila* in commercial soil and soil products. We asked the major manufacturers of bagged soil whether they had made recent changes in how garden soils were produced or if the composition of bagged soil products was different, but this was not confirmed. Further investigation of possible common routes of transmission through different soil products would be helpful to clarify the transmission pathway of *L.* non-*pneumophila* from bagged soil.

The increased incidence of *L.* non-*pneumophila* cases coincided with the gardening season and a possible factor behind the outbreak could be related to unusually warm conditions in Sweden in the spring and summer of 2018, which allowed the gardening season to start earlier. In May 2018, the average daily temperature in the Stockholm area was 2.2 °C higher than normal [24]. The warm weather may have facilitated earlier access to gardens, and longer or more frequent gardening, increasing the potential exposure time. It may also have caused soil to become dusty and dry, increasing the risk of inhaling dust particles and also enhancing *Legionella* growth in bagged soil. The warm weather would have increased the need for watering domestic gardens, increasing the potential opportunity to produce aerosols from soil. However, colleagues in other Nordic countries did not observe a comparable increased seasonal incidence of *L.* non-*pneumophila* cases, despite similar weather conditions.

A limitation of our analysis is that the outbreak was small, which may have affected the power of the study to detect true associations between exposures and illness. Some cases with severe LD could not be interviewed and are not included in the study, which may further limit our ability to detect associations. Both cases and controls were asked to recall activities during a period of several weeks before completing the questionnaire, introducing potential recall bias and lowering the chances of finding a true association: cases were more likely than controls to recall and report use of soil, with the risk of overestimating the association between soil exposure and illness.

Only six isolates from case specimens were cultured and further analysed by WGS, and the remaining samples were confirmed either by serology or FTD-PCR. As regional laboratories use different LD confirmation algorithms, cases with *L.* non-*pneumophila* infection might have been missed in regions with laboratories that do not use culture or PCR testing for *Legionella* spp. other than *L. pneumophila*. The fact that laboratories were encouraged to send samples of *L.* non-*pneumophila* for typing might have affected and inflated the numbers of cases with *L. longbeachae* compared with what has been reported previous years, but to what extent is difficult to estimate. Cases with *L.* non-*pneumophila* and therefore also *L. longbeachae* are certainly under-reported as other have described earlier [8,11]. Furthermore, there are limitations to the FTD respiratory 33 PCR kit used, as the manual for the kit states that the assay for *L. longbeachae* might also target *L. bozemanii* [25]. This implies that among the 12 cases identified as *L. longbeachae* with this method, some might have been misdiagnosed and might in fact have been *L. bozemanii.* However, given the polyclonality of *L. longbeachae* in human and soil samples, such misdiagnosis would lead to the same conclusion regarding risk factors for infection with *L.* non-*pneumophila.*

In conclusion, although we were not able to confirm an exact source for this outbreak of *L.* non*-pneumophila* and particularly *L. longbeachae,* the case–control study indicated that handling soil was associated with disease; the isolation of *L. longbeachae* from different soils from cases’ homes or gardens provided further evidence that handling soil is a risk for contracting *L.* non*-pneumophila* infections.

### Lessons learned

The findings from this study suggest that it is unlikely to find a genetic match between a soil specimen and a human specimen because of the wide polyclonality of *L. longbeachae* in soil samples. Therefore, WGS may be of limited use to confirm a specific soil as a vehicle of transmission of infection with *L. longbeachae,* and additional epidemiological investigations are needed to support appropriate public health measures.

A diagnosis of LD should be considered for individuals with community-acquired pneumonia who report gardening and using bagged soil before onset of disease. If this transmission route is suspected, additional diagnostic methods, other than the *L. pneumophila*-specific UAT, are required for characterisation and detection.

Individuals who are at increased risk for LD due to compromised immunity or underlying health conditions should be advised about the potential risk of infection with *Legionella* from soil products. How to disseminate this information needs to be investigated.
